# Vimentin Suppresses Inflammation and Tumorigenesis in the Mouse Intestine

**DOI:** 10.3389/fcell.2022.862237

**Published:** 2022-03-25

**Authors:** Linglu Wang, Ponnuswamy Mohanasundaram, Michelle Lindström, Muhammad Nadeem Asghar, Giulia Sultana, Julia O. Misiorek, Yaming Jiu, Hongbo Chen, Zhi Chen, Diana M. Toivola, Fang Cheng, John E. Eriksson

**Affiliations:** ^1^ School of Pharmaceutical Sciences (Shenzhen), Shenzhen Campus of Sun Yat-sen University, Shenzhen, China; ^2^ Cell Biology, Biosciences, Faculty of Science and Engineering, Åbo Akademi University, Turku, Finland; ^3^ Department of Molecular Neurooncology, Institute of Bioorganic Chemistry Polish Academy of Sciences, Poznan, Poland; ^4^ Key Laboratory of Molecular Virology and Immunology, The Center for Microbes, Development and Health, Institut Pasteur of Shanghai, Chinese Academy of Sciences, Shanghai, China; ^5^ University of Chinese Academy of Sciences, Beijing, China; ^6^ Faculty of Biochemistry and Molecular Medicine, University of Oulu, Oulu, Finland; ^7^ Turku Center for Disease Modeling, University of Turku, Turku, Finland; ^8^ InFLAMES Research Flagship Center, Åbo Akademi University, Turku, Finland

**Keywords:** vimentin, colon, dextran sodium sulfate, inflammation, tumorigenesis

## Abstract

Vimentin has been implicated in wound healing, inflammation, and cancer, but its functional contribution to intestinal diseases is poorly understood. To study how vimentin is involved during tissue injury and repair of simple epithelium, we induced colonic epithelial cell damage in the vimentin null (Vim^−/−^) mouse model. Vim^−/−^ mice challenged with dextran sodium sulfate (DSS) had worse colitis manifestations than wild-type (WT) mice. Vim^−/−^ colons also produced more reactive oxygen and nitrogen species, possibly contributing to the pathogenesis of gut inflammation and tumorigenesis than in WT mice. We subsequently describe that CD11b^+^ macrophages served as the mainly cellular source of reactive oxygen species (ROS) production *via* vimentin-ROS-pSTAT3–interleukin-6 inflammatory pathways. Further, we demonstrated that Vim^−/−^ mice did not develop colitis-associated cancer model upon DSS treatment spontaneously but increased tumor numbers and size in the distal colon in the azoxymethane/DSS model comparing with WT mice. Thus, vimentin has a crucial role in protection from colitis induction and tumorigenesis of the colon.

## Introduction

Abundant evidence reveals links between wound healing and cancerous tumor growth in a variety of common epithelial tumors (Dvorak, 1986) (Ben-Neriah and Karin, 2011). One good example is inflammatory bowel disease (IBD). Patients suffering from IBD with tissue damage and chronic mucosal inflammation are predisposed to the development of colorectal cancer (CRC), one of the most common malignancies in the world (Grivennikov et al., 2010a; Parent et al., 2010; Xie et al., 2020). Various cytokines and chemokines promote a localized inflammatory response upon intestinal injury and alter proliferation or survival of premalignant cells, thereby promoting oncogenesis (Newkirk et al., 2007).

Vimentin, the major type III intermediate filament protein prominently expressed throughout mesenchymal cell types, is strongly upregulated following injury to various tissues (Ivaska, 2011; Satelli and Li, 2011; Gan et al., 2016). Vimentin acts as a signal scaffold and functional determinants for many signaling pathway involved in cell migration during wound repair (Chernoivanenko et al., 2013; Chung et al., 2013). Loss of vimentin leads to delayed wound healing due to impaired directional migration and contraction (Eckes et al., 1998; Eckes et al., 2000; Ivaska et al., 2007; Dave and Bayless, 2014). Vimentin in mesenchymal repair cells is associated with myosin IIB and modulates the collective migration of the lens epithelium in response to wounding (Menko et al., 2014). Induction of vimentin by the TGFβ1-Smad pathway in alveolar epithelial cells is a requisite for wound repair after lung injury (Rogel et al., 2011).

Vimentin has been indicated to modulate cell fate and cellular function of immunocytes. For instance, vimentin is cleaved by caspases in apoptotic neutrophils and is then found to be expressed on the surface of the apoptotic neutrophils (Lavastre et al., 2002). This may be a recognition signal of apoptotic neutrophils by phagocytes for the inflammatory resolution (Moisan and Girard, 2006). Pronounced reorganization of the vimentin network during circulating lymphocyte extravasation is a primary structural supporting source of lymphocytes, which limits mechanical deformation of the cells upon chemokine-induced polarization and stimulates cells migrating through size-limited endothelium pores (Brown et al., 2001). N-terminal phosphorylation and reorganization of vimentin by the PI3Kγ signaling pathway are necessary for chemokine-induced transmigration of leukocytes to the inflammation sites (Barberis et al., 2009).

The importance of vimentin in inflammation and the immune response became more evident from several studies on the vimentin knockout (Vim^−/−^) mouse model. Although Vim^−/−^ mice develop and reproduce without any devastating defects, it was found that they have leaky endothelial vessels and disrupted homing of lymphocytes (Nieminen et al., 2006). Interestingly, there is disturbed distribution of the adhesion molecules in both migrating and the receiving cells, such as integrins in the lymphocytes and intercellular cell adhesion molecule-1 and vascular cell adhesion molecule 1 in endothelial cells. The lack of vimentin either in lymphocytes or in endothelial cells severely impaired transcellular migration of lymphocytes through endothelial cell barriers (Nieminen et al., 2006).

Interestingly, vimentin may also participate in the pro-inflammatory responses required for elimination of bacterial infections, especially the production of reactive oxygen species (ROS) and nitric oxides species from macrophages upon epithelial injury of the gut (Mor-Vaknin et al., 2013; Mahesh et al., 2016). Recently, vimentin was found to directly interact with the inflammasome NLRP3 and regulate its activation in acute lung injury models. Loss of vimentin inhibits the activation of NLRP3 inflammasome signaling and cytokine production and thereby inhibits the pathophysiologic events upon injury, including lung inflammation, leaky endothelial, and alveolar epithelial barriers (Dos Santos et al., 2015; Zhou et al., 2020). However, different results in murine air pouch model suggested that vimentin is dispensable to establish an acute inflammatory response *in vivo*, indicating that the specificity of vimentin in regulation of inflammation is likely to be different *in vivo* different models (Moisan et al., 2007; Pan et al., 2021), probably due to complex crosstalk among microbiota, organic barriers, and immune system in the wound repair.

In our recent study, by integrating defined *in vitro* and *in vivo* models of epidermal wound healing, we found that vimentin IFs coordinate balanced signals regulating fibroblast proliferation and epithelial-to-mesenchymal transition, two significant cellular activities in wound repairing process. The absence of vimentin inhibited these cellular processes, causing a delayed wound re-epithelialization and chronic inflammation in the injured lesions (Cheng et al., 2016). Therefore, we are interested whether loss of vimentin induces chronic inflammation upon injury of other types of epitheliums, such as the intestine, and whether it would provide a permissive environment for tumor onset in the colon.

To test this hypothesis, we applied a well-known dextran sodium sulfate (DSS)–induced colitis model and an azoxymethane (AOM) plus DSS colitis-associated cancer (CAC) model to Vim^−/−^ mice. We showed that the deletion of vimentin increases the level of ROS derived from macrophages *via* the vimentin-ROS-pSTAT3–interleukin-6 (IL-6) inflammatory pathways. Furthermore, Vim^−/−^ mice have increased tumor size and number in the distal colon comparing with WT mice, which indicates that vimentin has a crucial role in colitis induction and tumorigenesis of the colon.

## Results

### Increased Persistent Inflammation in Vimentin Null Mice During Chemical Intestinal Tissue Damage

**FIGURE 1:**
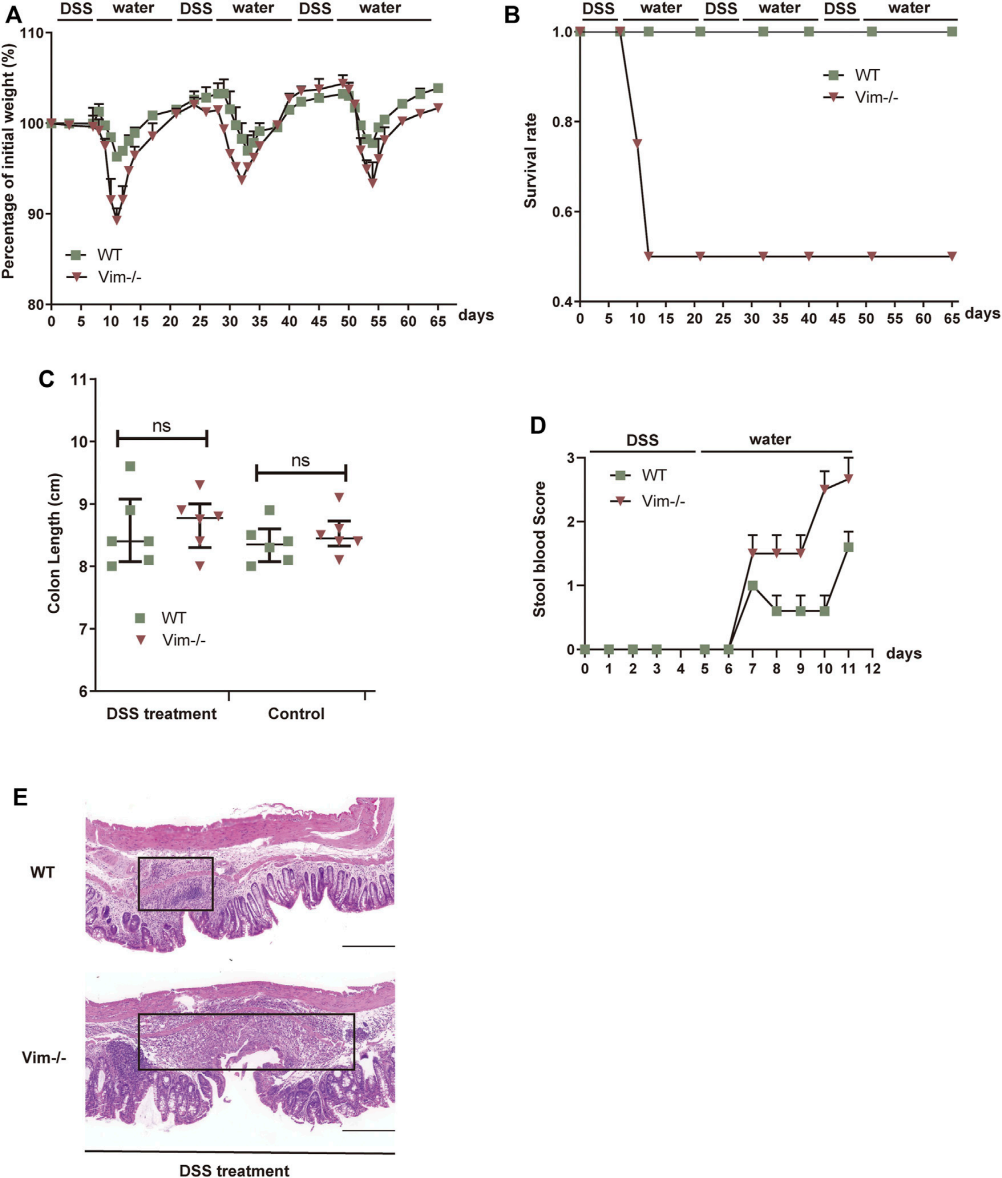
Lack of vimentin promotes DSS-induced colitis. **(A–C)** Vimentin null (Vim^−/−^) and wild-type (WT) mice were fed with 2.5% DSS or water for 7 consecutive days and then maintained with water for 14 days for three cycles. The mice were monitored for **(A)** body weight loss, **(B)** survival rate, and **(C)** total colon length after sacrificing at day 65. **(D)** Vim^−/−^ and WT mice were fed with 2.5% DSS for 5 consecutive days, and stool blood scores from day 0 to day 11 were assigned as follows: 0, no blood; 1, positive hemoccult; 2, visible traces of blood; 3, gross rectal bleeding. **(E)** Representative pictures of Hematoxylin and Eosin staining of WT and Vim^−/−^ mouse colon at day 65 upon 3× DSS induction or untreated ones (Control). Scale bar, 500 μm. Black box indicates the inflamed tissue area. *n*= 6, bars = mean ± SEM in all figure graphs; *, *p* < 0.05; **, *p* < 0.01; ***, *p* < 0.001.

First, we employed an intestine wound model to explore whether the delayed wound healing and prolonged inflammation phenotype that we observed during skin tissue damage with loss of vimentin (Cheng et al., 2016) can be recapitulated in a simple epithelium. In this chronic colitis model, 2.5% dextran sulfate sodium (DSS) is toxic to the colonic epithelial cells and induces tissue damage. After DSS-induced injury for 7 days, the mice are given 14 days of a water regimen to repair the colon epithelium, and this process is repeated to form three injury-healing cycles. In this model, following every round of wounding of the colon, the peak of disease (rectal bleeding, diarrhea, and weight loss) occurred during each healing period. Consistent with our hypothesis, Vim^−/−^ mice subjected to DSS treatment exhibited a more dramatic weight loss, a higher rate of mortality, and increased blood in the stool (Figures 1A–D). In contrast, WT mice exposed to the same treatment had only mild weight loss, minor levels of blood in the stool, and no mortality (Figures 1A–D), indicating loss of vimentin inflames intestine upon injury. In line with these data, histologic images from colon sections of Vim^−/−^ mice showed severe ulceration in gut mucosal lining, compared to WT (Figure 1E). Thus, these data support our hypothesis that endogenous vimentin is involved to a prompt colonic repair and to alleviate the injury-induced inflammation program.

### Lack of Vimentin Increases ROS/RNS–NF-κB–IL-6 in the Colon Primarily by Macrophages

**FIGURE 2:**
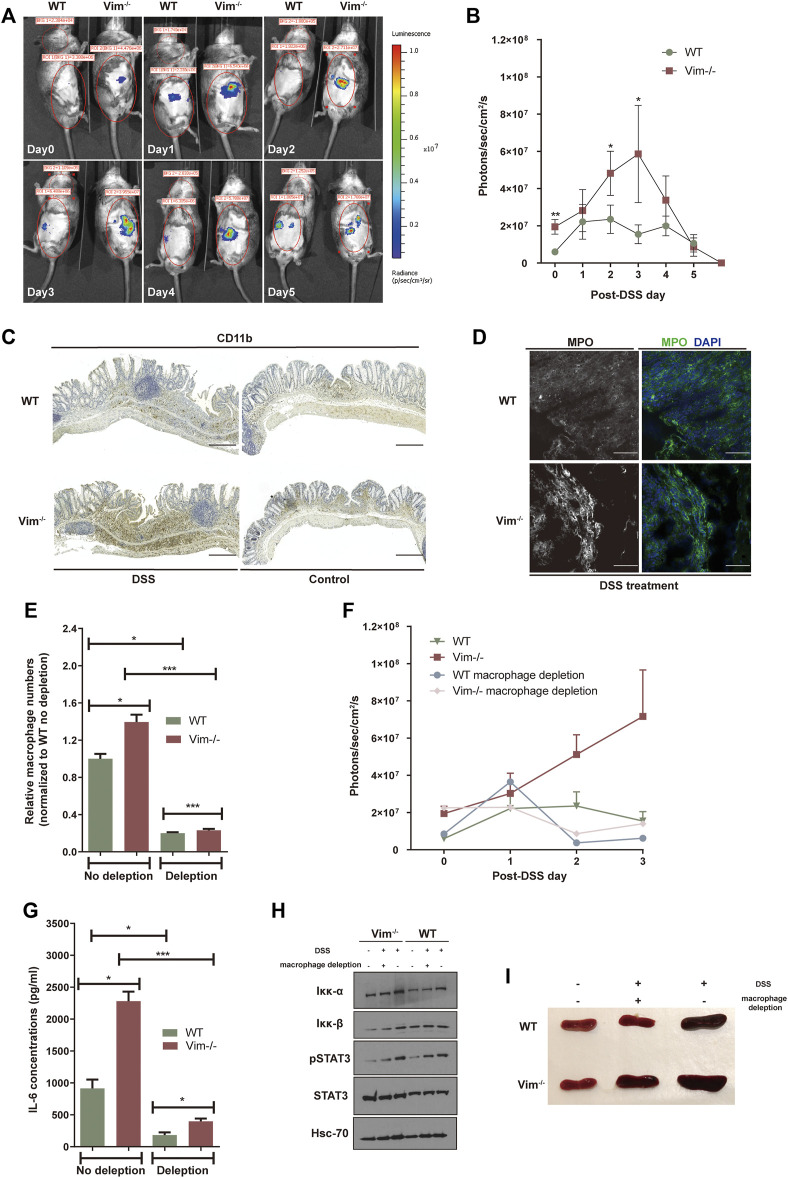
Lack of vimentin increases ROS in the colon primarily by macrophages. **(A,B)** DSS (2.5%) were administered to Vim^−/−^ and WT mice for 7 consecutive days from - day 1. Luminescent images **(A)** were taken from day 0 to day 5 upon DSS treatment after injection with L-012 and average luminescence values **(B)** were counted. Bars = mean ± SEM, *n* = 12. The pseudo colors represent photons/s cm^2^ sr. time dependency of the L-012 luminescent signal. **(C,D)** DSS (2.5%) were administered to Vim^−/−^ and WT mice for 7 consecutive days, followed by 8 days off water without DSS. Representative images and quantitation of **(C)** CD11b-labeling and **(D)** MPO-labeling of the colon samples on day 15 of the experiment. Scale bars, 500 μm. In panels B and D, bars = mean ± SEM, *n* = 6. **(E)** Peritoneal macrophage numbers in DSS-induced WT and Vim^−/−^ mice upon depletion of macrophages in the circulation using clodronate liposomes or control liposomes. Bars = mean ± SEM, *n* = 3. **(F)** *In viv*o luminescence values in different timepoints of mice injected with L-012 upon DSS treatment and macrophage depletion. Bars = mean ± SEM, *n* = 3. **(G)** IL-6 concentration in the circulation of DSS-induced WT and Vim^−/−^ mice upon macrophage depletion. Bars = mean ± SEM, *n* = 3. **(H)** Immunoblotting of Iκκ-α, Iκκ-β, pStat3, Stat-3, and Hsc-70 expression of total colon tissue lysates. **(I)** Representative spleen size of DSS-induced WT and Vim^−/−^ mice.

Previous work showed that vimentin modulates the production of ROS and nitrogen species (RNS), an important part of tissue damage and the inflammatory response (Tolstonog et al., 2001; Zhang et al., 2001). To address whether loss of vimentin could exacerbate the oxidative stress in the mouse colon upon DSS wounding, we used the chemiluminescent probe L-012, a ROS/RNS-sensing probe (Kielland et al., 2009; Asghar et al., 2014) for noninvasive imaging of ROS/RNS production in living mice. L-012 can be widely distributed and spatially and temporally emit light responding to inflammation sites. The L-012–indicating luminescence was later recorded by an ultrasensitive CCD camera (IVIS Spectrum, Xenogen, CA, USA). Strong luminescent signals observed from colon regions corresponded to inflammation in WT and Vim^−/−^ colon regions, which peak on 3 days post 2.5% DSS oral administration (Figures 2A,B). However, Vim^−/−^ mice were found to release higher levels of luminescence signals and thus to produce significantly more ROS/RNS than that WT mice in response to stimulation with DSS (Figures 2A,B). Thus, vimentin is crucial for regulating ROS/RNS upon DSS-induced tissue damage.

We next aimed to identify the cellular source of ROS and downstream inflammatory signaling in the colon. DSS is toxic to the epithelial cells in the crypts of the colonic mucosa and is commonly used to induce tissue damage *via* the mediation of inflammation (Wirtz et al., 2007). Therefore, upon DSS stimulation, ROS in the intestines may derive from macrophages and regional microflora (Formentini et al., 2017). Identified by anti-CD11b staining, we found that myeloid cells became detectable in colon sections of both WT and Vim^−/−^ mice already 7 days after DSS induction, followed by the appearance of localized inflammatory cells in histology on day 15 (Figure 2C). Furthermore, we also observed more CD11b^+^ monocytes or macrophages infiltrated in KO colon lamina propria (Figure 2C). Correspondingly, myeloperoxidase (MPO), an important enzyme with phagocytic lysis activity secreted by monocytes, exhibits strong cytoplasmic signals in Vim^−/−^ colons compared to the WT group (Figure 2D). Thus, monocytes/macrophages were more abundant in the Vim^−/−^ leukocyte population than in the WT population, and their overall contribution to phagocytic enzyme MPO production was greater (Figure 2D).

We next asked whether macrophages would be necessary for this inflammatory signaling. We found depletion of macrophage in the circulation using clodronate liposomes largely inhibited the peritoneal macrophages numbers in DSS-induced WT and Vim^−/−^ mice to a similar level (Figure 2E). Interestingly, depletion of macrophages has significantly impaired the upregulation of ROS/RNS-dependent luminescent signal *in vivo* (Figure 2F), suggesting that macrophages are the major cell component generating ROS/RNS during DSS-induced inflammation.

As shown in Figure 2F, after macrophage depletion, the peak level of ROS/RNS was reduced, compared with the no-depletion control group in Vim^−/−^ mice. These data suggest that the accumulation of ROS/RNS in the wound of Vim^−/−^ mice after intestinal injury mainly results from the recruited macrophages. This was consistent with previous studies showing that monocytes lacking vimentin produce more superoxide and nitric oxide and thereby activate several intracellular signaling cascades that lead to proinflammatory genes activation (Mor-Vaknin et al., 2013).

Furthermore, it is likely that resident macrophages or macrophages transported to the inflammation site could be responsible to produce proinflammatory cytokines in these lesions. In line with this hypothesis, analysis of cytokine productions in circulation demonstrated a significantly reduced level of IL-6 concentration in Vim^−/−^ and WT group upon macrophage depletion, although Vim^−/−^ mice still maintains a relatively higher level of IL-6 concentration than WT group after macrophage depletion (Figure 2G). Consistently, immunoblot analysis of total colon tissue lysates revealed the substantial increase of STAT3 phosphorylation and NF-κB induction in DSS-treated WT and Vim^−/−^ mice (Figure 2H). Furthermore, Vim^−/−^ mice develop stronger splenomegaly than WT upon DSS induction, and we found that systemic depletion of macrophages prevented the development of splenomegaly in these mice (Figure 2I). Thus, enhanced cytokine secretion by macrophages, at least in part, induced Vim^−/−^ mice from DSS-induced colitis.

### Deletion of Vimentin Increases Colitis-Associated Tumor Incidence

**FIGURE 3:**
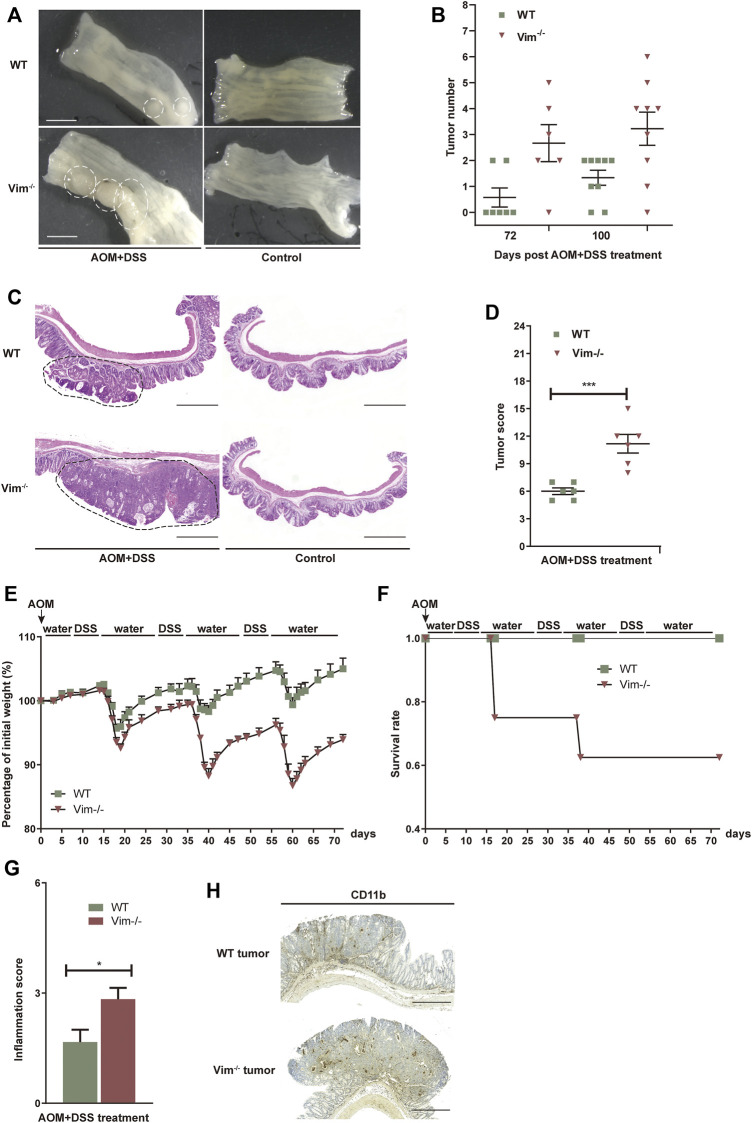
Increased tumorigenesis in vimentin null mice in a colitis-associated colon cancer model. **(A)** Representative macroscopic view of WT and Vim^−/−^ mouse colon on day 75 upon AOM + DSS induction. Scale bar, 1 mm. **(B)** Colitis-associated colorectal was induced *via* injection of AOM (7.5 mg/kg) and three cycles included 2% DSS feeding for 7 days and water for 14 days. The tumor numbers per mouse colon between WT and Vim^−/−^ mice on day 75 and day 100 were recorded cumulatively from three independent experiments. Each dot represents one mouse. Lines indicate mean ± SEM. *n* = 24 **(C)** Representative pictures of Hematoxylin and Eosin staining of WT and Vim^−/−^ mouse colon on day 100 upon AOM + DSS induction or untreated ones (Control). Scale bar, 500 μm. **(D)** Tumor score between WT and Vim^−/−^ colons. *n* =6. **(E,F)** Vim^−/−^ and WT mice during AOM + DSS treatment in colitis-associated colon cancer model were monitored for body weight loss and the survival during the 72 days of treatment. **(G)** These mice from colitis-associated colon cancer model were sacrificed at day 72 and histological scores of colon tumor sections were determined on the basis of the colitis index. **(H)** Representative images and quantitation of CD11b-labeling of the colon tumor samples.

To test whether the deletion of vimentin is prone to chronic injury-associated cancer, we introduced a AOM plus DSS colitis-associated colorectal model to resemble the pathology of human colitis-associated neoplasia (Tanaka et al., 2003; Becker et al., 2004). In this model, tumor initiation depends on metabolic activation of AOM, a colon-specific carcinogen (Neufert et al., 2007) and tumor development is promoted by DSS induced tissue damage and inflammation. To support our hypothesis, tumor development was strongly accelerated with significantly increased tumor number and size in Vim^−/−^ mice comparing to their wild-type (WT) counterparts (Figures 3A,B). For up to 75 days of treatment (12 days after the last treatment cycle), very few neoplastic lesions were observed in the WT colons by histopathological examination. However, by the same time, low-grade dysplastic lesions were apparent the majority of Vim^−/−^ mice and, correspondingly, in average tumor load, the diameters of all tumors in a given mouse (Yilmaz and Christofori, 2009) was significantly higher in Vim^−/−^ mice (Figures 3A,B). The absence of vimentin had a stronger effect on tumor number and size 100 days upon the treatment (Figure 3B). By day 100, multifocal adenoma was seen in both Vim^−/−^ and WT colons, encompassing epithelium areas with low- and high-grade flat dysplasia. Notably, tumor morphology was similar between Vim^−/−^ and WT control mice (Figure 3C). Throughout the entire period, no invasive carcinoma (the cancerous glands penetrated the submucosa or desmoplastic reaction of the surrounding stroma) were detected in any WT or Vim^−/−^ mouse. Gross assessment of the colon tumors by histopathological examination also revealed a more severe tumor score (Figure 3D) in Vim^−/−^ mouse. Interestingly, in mice treated with AOM only, no tumors were observed in both WT and Vim^−/−^ mice (Supplementary Table S1), suggesting that, in our mouse model, inclusion of a chemical carcinogen is not enough to switch the response toward a different course with pathological and molecular features commonly observed in sporadic human CAC. Besides, we found that consistent with the chronic colitis model, in the colitis-related cancer model, Vim^−/−^ mice also exhibit more severe inflammatory symptoms (Figures 3E–G) than WT mice. Inflammatory cells, which often infiltrate tumors and preneoplastic lesions, produce a variety of cytokines and chemokines that propagate a localized inflammatory response (Pikarsky et al., 2004; Stojadinovic et al., 2008). We found that CD11b^+^ macrophages were accumulated in tumor regions of WT and Vim^−/−^ colon cancer samples (Figure 3H) similar to after DSS treatment in Figure 2C, suggesting that loss of vimentin not only may increase susceptibility to inflammation but also may greatly accelerate inflammation-associated colon cancer. Together, these data indicate that vimentin is a negative regulator for both tumor initiation and tumor growth in CAC. Vimentin deficiency leads to accelerated and increased tumorigenesis in this colitis-associated colon cancer model.

### Increased Proliferation and Tumor Grade in Vim^−/−^ Cancer

**FIGURE 4:**
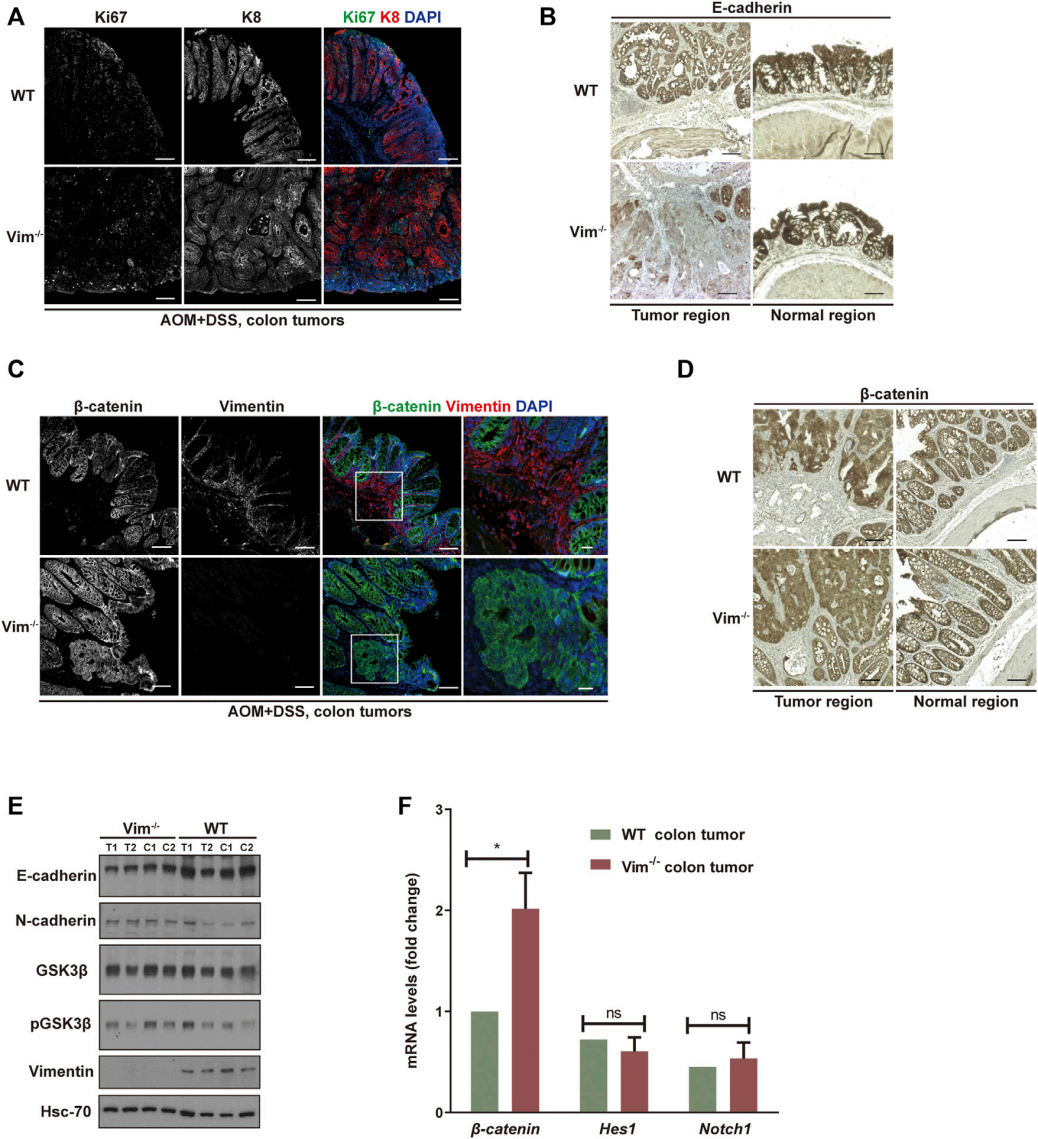
Increased proliferation and tumor grade in Vim^−/−^ cancer. **(A)** Representative confocal images indicated the expression of ki67 (in green), K8 (in red), and DAPI (in blue) in WT and Vim^−/−^ colon tumors upon AOM and DSS treatment. **(B)** Representative pictures of tumors and their neighboring normal regions by immunohistochemical labeling of E-cadherin of WT and Vim^−/−^ mouse colon upon AOM + DSS induction. **(C)** Representative confocal images of the expression of β-catenin (in green), vimentin (in red), and DAPI (in blue) in WT and Vim^−/−^ colon tumors upon AOM and DSS treatment. **(D)** Representative pictures of tumors and their neighboring normal regions by immunohistochemical labeling of β-catenin of WT and Vim^−/−^ mouse colon upon AOM + DSS induction. Scale bar (A–D), 200 μm. **(E)** Extracts (30 μg) from colon tumors of WT and Vim^−/−^ mice were immunoblotted with anti–E-cadherin, anti–N-cadherin, anti–GSK-3β, anti–P-GSK-3β, or anti-vimentin. Hsc-70 blotted from the lysates to control for equal loading. **(F)** Quantitative real-time PCR (qRT-PCR) analysis of transcripts for β-catenin, Hes-1, and Notch-1 in WT and Vim^−/−^ mouse colon tumors. Error bars = ± SEM; *n* =6; *, *p* < 0.05; ns, not significant.

We further assessed colon neoplasia lesions characterized by several features of incipient malignant disease, including irregular architecture/expansion of the colonic crypts, co-expression of epithelial and mesenchymal markers, and activation of β-catenin. We confirmed the loss of vimentin expression in Vim^−/−^ colons *via* immunofluorescence and Western blotting analysis (Figures 4C,E). In addition, the analysis showed that the tumors come from the intestinal epithelium indicated by intact epithelial keratin 8 (K8) expressions in WT and Vim^−/−^ tumor lesions in mice (Figure 4A). Proliferation marker Ki67 labeling suggests that the proliferation rate of tumor cells was higher in Vim^−/−^ mice than WT mice (Figure 4A), but no obvious difference in basal crypt proliferation was revealed between control WT and Vim^−/−^ mice. This was accompanied with the down-modulation of E-cadherin, a differentiation marker important for epithelium barrier function in Vim^−/−^ tumors (Figure 4B). Parallel Western blotting analysis confirmed decreased E-cadherin and slight upregulation of N-cadherin in tumor regions of Vim^−/−^ mice (Figure 4E). Wnt pathway activation is markedly frequent in human CAC, mostly involving early adenomatous polyposis coli mutations (Krieglstein et al., 2001). Notch pathway is another common signaling pathway involved in CAC development. Remarkably, regardless of genotype, nuclear and cytoplasmic β-catenin accumulation predominated in these tumors (Figures 4C,D), consistent with the frequent induction of β-catenin transcripts in Vim^−/−^ tumors (Figure 4F), which is indicative of robust Wnt pathway activation. In contrast, in WT tumors, β-catenin was retained at the plasma membrane, suggesting a relative inactive Wnt pathway in these WT neoplasms. No obvious changes in Notch signaling were detected on the basis of the qPCR analysis of Notch1 and its target gene Hes1 between WT and Vim^−/−^ tumors (Figure 4F). In sum, both histologically and molecularly, the DSS mouse model of IBD-associated cancer closely reproduces features frequently observed in its human counterpart, and there is an increased proliferation and tumor grade in Vim^−/−^ cancer.

## Discussion

CRC is a multifactorial human disease that develops slowly over time as a result of inflammation and/or accumulation of mutations (Roper et al., 2013). We postulated upon our data that vimentin is a potent factor to participate in maintaining the homeostasis of cell and tissue and, in doing so, protect colonic epithelial tissues from inflammation and cancer and further may facilitate intestinal repair. The increased tumor burden of Vim^−/−^ mice in the colitis-associated caner model is due to a delayed wound healing and the increased susceptibility to DSS-induced chronic colitis. This is in line with the stress-protective functions of intermediate filaments proteins particularly under regenerative conditions in many tissues (Toivola et al., 2010).

No tumors developed in the non-inflammatory AOM cancer model in Vim^−/−^ mice, which is consistent with the earlier report that the absence of vimentin has no discernible effect on tumorigenesis in a teratocarcinoma model (Colucci-Guyon et al., 1994). Our data indicates that Vim^−/−^ mice are more susceptible to DSS-induced colitis, and the chronic inflammation microenvironment may be a key tumor promoter. Vimentin has been reported to promote, to inhibit, or to have no effect in inflammation in different tissue injury models, indicating the context and model specificity of vimentin (Mor-Vaknin et al., 2003) (Mor-Vaknin et al., 2013) (Moisan et al., 2007) (Dos Santos et al., 2015). Furthermore, using the subcutaneous air pouch model in the back of mice, the Vim^−/−^ mouse appears to have normal acute inflammatory response to lipopolysaccharide (LPS) or IL-21 stimulation (Moisan et al., 2007), indicating the specificity of vimentin regulation of inflammation in different *in vivo* models.

Vimentin was recently shown to be involved in the experimental murine colitis (Mor-Vaknin et al., 2013). However, their findings from earlier results suggest that vimentin secreted by activated human macrophages participates in the bacterial killing and the generation of oxidative metabolites (Mor-Vaknin et al., 2003). We found that, in the same experimental acute colitis model, Vim^−/−^ mice are likely more capable to mediate bacterial killing by abundant production of ROS and nitric oxides from macrophages (Mor-Vaknin et al., 2013). In addition, increased ROS and other oxidative damage may stimulate gut inflammation and the development of IBDs, but these Vim^−/−^ mice exhibited less gut inflammation and intestine disease (Mor-Vaknin et al., 2003). Therefore, we suspect that the complex crosstalk between microbiota, intestinal barrier, and immune system in the wound repair may vary from one laboratory from another (Peterson and Artis, 2014).

Inflammatory responses play decisive roles at different stages of tumor development. Many environmental causes of cancer and risk factors are associated with some form of chronic inflammation. Prolonged exposure of irritants in some organs, such as DSS in colon, prepares an inflammatory breeding ground for tumor development, since chronic inflammatory responses can promote tumor progression and metastatic spread, cause local immunosuppression, and further augment genomic instability (Grivennikov et al., 2010b). Our study demonstrated that vimentin knockout enhance inflammatory responses, such as the accumulation of ROS and the recruitment of CD11b^+^ macrophages and monocytes in the colon. Brought with this enhancement, inflammation-induced mutagenesis and tumor suppression may occur in the injured colon, which benefit tumor initiation. Once initiated cells appear, the depletion of vimentin may break the balance of inflammation-associated protumorigenic and antitumorigenic effects, thus promoting the tumor growth. In this process, some signaling pathways intimately involved are activated, such as Wnt/GSK3β/β-catenin pathways. Our study showed a slight increase of GSK3β phosphorylation, which inhibits GSK3β activity as a kinase and decreased the phosphorylation of its targeted protein, β-catenin, in the tumor of vimentin null mice. This reduced phosphorylation provided β-catenin from being ubiquitinated and regraded in proteasomes and further induced β-catenin accumulation. Other pathways including NF-κB and STAT3 signaling accelerated the transcription of β-catenin, thus resulting in a high level of β-catenin. The accumulation subsequently would induce epithelial mesenchymal transition and promote tumor invasion.

**FIGURE 5:**
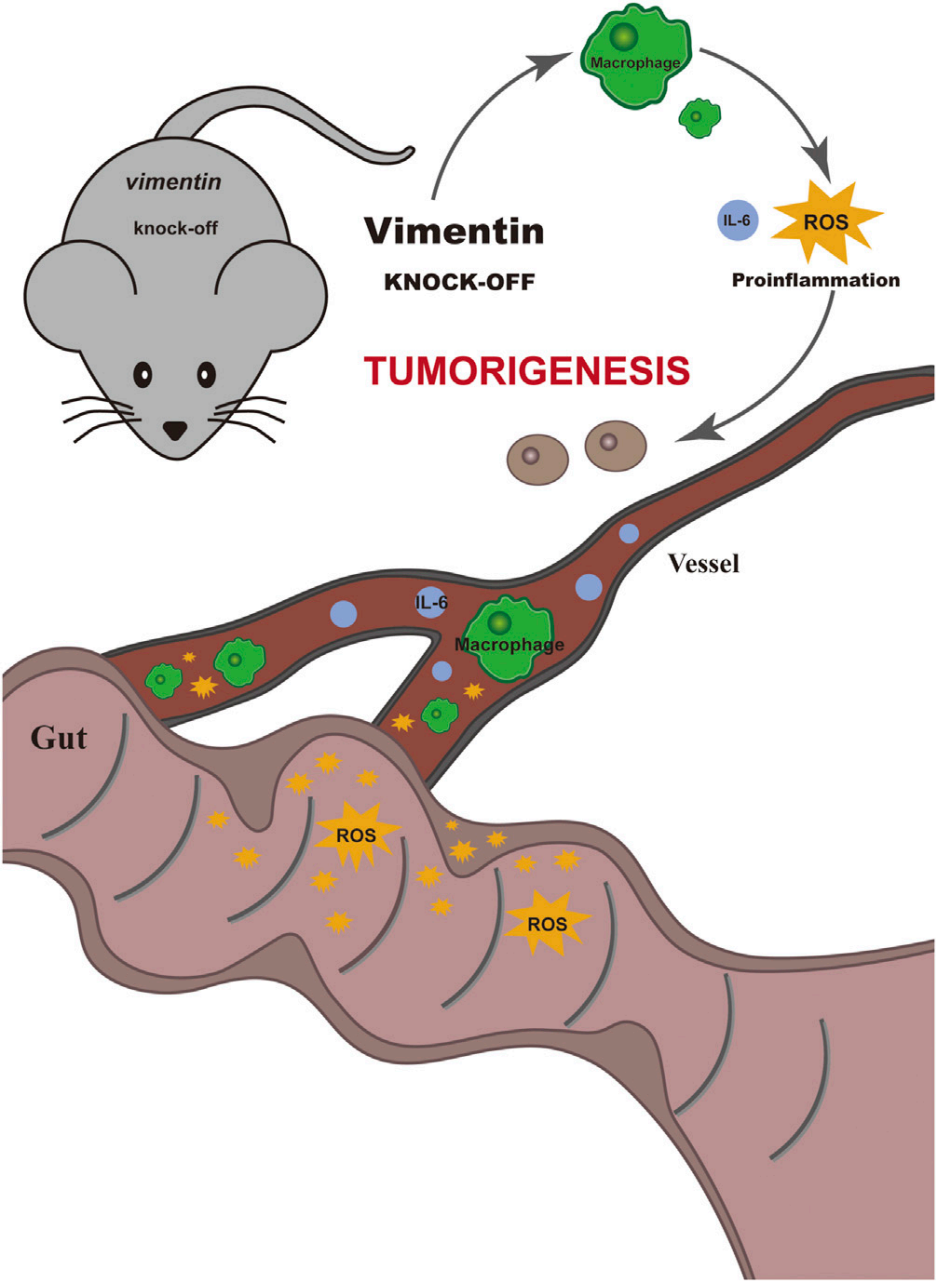
Schematic illustrate the mechanism of vimentin protecting the intestinal epithelium from inflammation and promoting tumorigenesis. The loss of vimentin *in vivo* leads to susceptibility to develop colitis-associated colorectal cancer upon the combination of AOM carcinogen treatment and DSS inflammatory injury, whereas the deletion of vimentin alone does not predispose to colitis-associated colorectal cancer.

In summary, our data indicate that vimentin is a factor important for protecting the intestinal epithelium from inflammation and tumorigenesis promotion. We showed here that the loss of vimentin *in vivo* leads to susceptibility to develop colitis-associated CRC upon the combination of AOM carcinogen treatment and DSS inflammatory injury, whereas the deletion of vimentin alone does not predispose to colitis-associated CRC (Figure 5).

## Materials and Methods

### Animals

All animals involved in studies are reported in accordance with the ARRIVE (Animal Research: Reporting of *In Vivo* Experiments) guidelines for reporting experiments involving animals (Kilkenny et al., 2010; McGrath et al., 2010). The specific pathogen–free mice are maintained at the Central animal Laboratory at University of Turku under permit 7284/04.10.03/2012 of the Ethical Committee for Animal Experiments of the University of Turku. Animal were kept under standard conditions during the whole experiments. Vimentin heterozygous mice with a mixed background of C57BL/6 and SVJ/129 were used to generate vimentin deficient homozygotes (Vim^−/−^) and WT offspring (Colucci-Guyon et al., 1994). The genotypes of the mice were determined by PCR genotyping methods (Truett et al., 2000). Ten- to 12-week-old mice were used for the experiments. Mice in experiments including controls were cohoused littermates. Animal experiments were performed under the approved animal study protocols 2893/04.10.03/2011 and 2007-07005 by the State Provincial Office of South Finland and housed in Turku Central Animal facility, Finland.

### Experimental Procedures and Animal Weight

For the AOM plus DSS colitis-associated colorectal model, briefly on day 0, mice were injected intraperitoneally (i.p.) with a single dose of AOM (7.5 mg/kg; Sigma) and then maintained on regular diet and water for 7 days. On day 7, mice were fed with 2% dextran sulfate sodium (DSS; TdB Consultancy; dissolved in water) for 7 days and maintained on autoclaved water for 14 days as a cycle. This 2% DSS-autoclaved water treatment cycle was conducted for two more times later (Wirtz et al., 2007).

For the AOM CRC model, the mice were injected i.p. once a week with AOM (7.5 mg/kg) for 4 weeks and sacrificed.

For the chronic colitis model, mice were fed with 2.5% DSS for 7 days and later maintained on autoclaved water for 14 days for two more times.

Mouse body weights were monitored daily during DSS treatment and weakly during water treatment. The clinical course of disease was followed by changes of body weight and monitoring for signs of rectal bleeding or diarrhea. At the end of the experiment, the mice were sacrificed, and samples from small intestine and colon (proximal colon and distal colon) were collected for further analysis as described below.

### Tumor Scoring

Tumor scoring was analyzed with indicators as follows: mucosal ulceration; hyperplasia or colonic tumors; erosion, crypt loss, or abscess; colitis (inflammatory cells infiltration into the lamina propria mucosa); and edema. Severity scores ranged from 0 to 5 as follows: 0, within normal limits or absent; 1, minimal; 2, mild; 3, moderate; 4, marked; and 5, severe. Scores of every indicators were added together to get a total score as tumor scoring.

### Sample Collection and Analysis

Mice were sacrificed using carbon dioxide inhalation and subsequent cervical dislocation. Colon was opened lengthwise and divided into 4 parts. The colon samples were imaged by a Leica M60 microscope (Leica, Mannheim, Germany) for polyp or tumor counting. Macroscopic tumors (adenomas, polyps, or colonic aberrant crypt foci) were counted and measured with a caliper.

Tissue samples were collected from distal colon and snap frozen in liquid nitrogen for RNA extraction (RNAlater, Ambion) or protein extraction.

Colon samples were fixed in 4% paraformaldehyde in phosphate-buffered saline (PBS, pH 7.4) for 24 h, transferred to 70% ethanol, and processed for paraffin embedding and histological analysis. Colon was also embedded with Optimal Cutting Temperature compound (Sakura Finetek) for immunofluorescence staining.

### L-012 Probe Imaging

Mice were fed with 2.5% DSS for 7 consecutive days. At D1, mice were shaved and were imaged from D0 to D5. During bioluminescent, mice were narcotized with the anesthetic isoflurane (1.5–2.5%). The luminescent probe L-012 (Wako Chemical) was dissolved in sterile 0.9% NaCl to make the final working concentration of 50 mg/kg (Asghar et al., 2014). The imaging system used IVIS Spectrum (Xenogen, CA, USA).

### Hematoxylin and Eosin Staining, Immunofluorescence and Immunohistochemistry

For Hematoxylin and Eosin staining and immunofluorescence, colon samples were embedded in paraffin. The paraffin embedded samples were sectioned in 5 μm and deparaffinized and rehydrated subsequently. Samples stained with Hematoxylin and Eosin staining were used for histology analysis. For immunofluorescence staining of the cells and tissue sections (Cheng et al., 2011), antigen of sectioned samples was retrieved with 0.1M citrate buffer in pH 6.0. Sectioned samples were later blocked with 5% goat serum and were incubated with primary antibodies overnight at 4°C. Then, sectioned samples were stained with secondary antibodies at room temperature for 3 h. Finally, DAPI staining was conducted for 10 min.

For immunochemistry staining of tissue sections, sectioned samples were stained and visualized by ABC staining system (Vector lab) and later counterstained with Mayers hematoxyline (Histolab). Isotype counterpart was served as negative controls.

### Microscopic Image Acquisition and Quantification

All confocal images were acquired by Zeiss Zen software on a Zeiss LSM780 confocal laser scanning microscope (Carl Zeiss, Munich, Germany) with the following objectives: Plan-Apochromat 10× (NA of 0.45, air) and Plan-Apochromat 40× (NA of 1.30, oil). The following fluorochromes were used: Alexa Fluor 488 Dye (Invitrogen), Alexa Fluor 594 Dye (Invitrogen), and DAPI (Invitrogen). Immunohistochemistry images were taken with a Leica DC300F digital camera attached to a Leica DMLB microscope (Leica, Wetzlar, Germany). Images were viewed and adjusted with brightness and contrast by Adobe Photoshop software. Fluorescent images were processed and analyzed in a pipeline created in the BioimageXD framework (Kankaanpää et al., 2012). The pipeline detects the nuclei from the Hoechst channel and counts them. The cytoplasm of each cell was modeled with a 20-pixel ring around the nucleus. To quantify the invasion of neutrophils and macrophages in the colon tissues, sectioned samples embedded with paraffin from three different mice, and different time points per genotype were immunohistochemically stained for neutrophils (MPO) and macrophages (CD11b). Positively stained cells with distinct cellular borders and all cells presented within the sectioned samples were counted *via* a Zeiss Axiophot light microscope.

### Macrophage Isolation, Purification and Depletion

Mice were sacrificed by spinal dislocation and injected with 5 ml of PBS. Peritoneal macrophages acquired from ascites extraction were then isolated and purified by the MagniSort™ Mouse F4/80 Positive Selection Kit (Invitrogen). For macrophages depletion, the mice received an i.p. injection of 300 µl of clodronate liposomes (LIPOSOMA), 1 day before the experiment. Empty liposomes were used in the no-depletion group. Samples were collected 3–7 days after DSS treatment.

### ELISA Analysis

Peripheral blood serum samples were collected 3–7 days after DSS treatment, and IL-6 levels in the mouse serum were measured using an IL-6 ELISA kit (eBiosciences).

### Tissue Preparation and Western Blot Analysis

Tissue lysates were prepared by homogenizing intestines in ice cold protein lysis buffer (20 mM Tris pH 7.5, 150 mM NaCl, 2 mM EDTA, 1% Triton-X100, 10% glycerol) using a Tissue Lyser II (QIAGEN; Hilden, Germany) for 12 cycles of 30 s at 30 Hz. Protein levels of tissue lysates were normalized by a BCA kit (Thermo Fisher Scientific) (Weigmann et al., 2007). After lysed in SDS-PAGE loading buffer and boiled at 95°C for 5 min, proteins were separated by SDS polyacrylamide electrophoresis using 10% gel at 120 V for 90 min, and separated proteins were transferred in to nitrocellulose membrane by doing wet transfer at 100 V for 30 min. Membranes were later blocked with 5% milk and incubated with primary antibody at 4°C for overnight with shaking. Then, membranes were incubated with secondary antibody at room temperature for 1 h with shaking and proteins were detected by Amersham ECL reagent (Cytiva).

### Real-Time Quantitative PCR

Total RNA was extracted from colon tissue, tumors, colon biopsies, or cells using RNeasy mini kits (QIAGEN) or the FFPE Total RNA Isolation Kit (Invitrogen). cDNA was obtained by reverse-transcribing same amount of total RNA using the High-Capacity cDNA Reverse Transcription Kit (Applied Biosystems). The transcript levels of the genes were measured the SYBR Green PCR mix (Applied Biosystems) in an Applied Biosystems 7300 detection system (Bio-Rad). The quality of the quantitative PCR run was determined by standard curves and melting curve analysis. The data were normalized to the expression of a cellular housekeeping gene GAPDH. Primers sequences (forward and reverse) used in this study are listed in Supplementary Table S2.

### Statistical Analysis

The results are expressed as the mean ± SEM. Comparisons between two groups were analyzed by two-tailed t tests. Comparisons between multiple groups were analyzed by one-wayANOVA. *p* < 0.05 was considered significant. Statistical differences were calculated with the two-tailed unpaired *t*-test, and differences were considered significant at *p* ≤ 0.05. For statistical evaluation of qRT-PCR data, the values (logarithmic) were converted to ddCt values (linear log2 scale values), and *p*-values were calculated using one-tailed unpaired Student’s t-test.

## Data Availability

The original contributions presented in the study are included in the article/Supplementary Material, further inquiries can be directed to the corresponding authors.
